# Clinical Memoranda from the Surgical Clinic at the Sisters’ of Charity Hospital1Read before the New York State Medical Society, in Albany, February 7, 1894.

**Published:** 1894-04

**Authors:** Herman Mynter

**Affiliations:** Professor of Surgery, Niagara University, and Surgeon to the Sisters’ Hospital


### CLINICAL MEMORANDA FROM THE SURGICAL CLINIC AT THE SISTERS OF CHARITY HOSPITAL.[1]

By HERMAN MYNTER, M. D.,

Professor of Surgery, Niagara University, and Surgeon to the Sisters' Hospital.

During the last two years, I have had occasion to trephine twenty-seven times. Of these, the majority, fifteen in all, were made on account of fractures of the skull. They represent all forms of lesions, from compound and comminuted fractures extending into the base, with laceration of membranes and brain, to simple depressed fractures of the vertex. Four of these cases died, all of which had extensive fractures of the base of the skull.

Five cases were operated on account of traumatic epilepsy, four of which were greatly improved, although a cure was probably not obtained in any case.

Five cases were operated by linear craniectomy, for microcephalus with idiocy, without any apparent improvement in any case. One case was trephined for subdural hemorrhage with aphasia and hemiplegia, and recovered perfectly, and one case recovered, after trephining, from a brain abscess. All operations were performed with the usual precautions in the Sisters' hospital, and all recovered, with the exception of four with necessarily fatal injuries of the base of the skull. In regard to covering the bony defect after trephining, I have tried to implant a layer of leaf-gold unsuccessfully several times. Eretic granulations occurred on both sides of the gold, forming a semifluctuating, painful swelling, which necessitated its removal. I have twice successfully implanted a celluloid plate, covering the defect and inserted beneath the internal table.

In one case, I reimplanted successfully the whole bony disk removed by a very large (one and one-quarter inch) trephine. This is, of course, the ideal proceeding. In several operations for traumatic epilepsy I covered the dura, Mosaic fashion, with bits of the bone removed, without paying any particular attention to whether the internal table of the bits of bone was turned inwards or not. I thereafter closed the wound without drainage. All the cases, with the exception of one, in which suppuration occurred and the bone particles had to be removed, recovered without com-

1. Read before the New York State Medical Society, in Albany, February 7, 1894.

plication, and adhesion between the dura and the scalp was most successfully prevented. Where laceration of dura and brain was present, this method, of course, cannot be used, and in such cases I always pack the wound loosely with iodoform-gauze and expect healing by granulation.

Three of the cases deserve special mention :

### TREPHINING FOR SUBDURAL HEMORRHAGE WITH APHASIA AND RIGHT-SIDED PARESIS—RECOVERY.

Case XII.—Daniel Blackford, 25 years of age, entered the Sisters' hospital on November 13, 1893, with the following history : Twelve days previously, while in a fight, he was struck by his opponent's fist on the right side of the head and knocked down, striking the left side of his head on the plank sidewalk. He was able to walk home, but his face and eyes were swollen, and he complained of severe pain in the back of the head. Nothing further could be learned about his condition, while at home, except that he, on the day of his admission to the hospital, had had a fit, with convulsive movements of right side of face and arm. While being prepared for bed he had another fit with similar muscular contractions in face and arm.

On examination, paresis was seen of the right side of the face ; there was constant twitching of the muscles of the right hand and arm, tongue drawn toward the left side, left pupil contracted. He experienced some sensation in the arm and leg, but, apparently, none in the face. There was some paresis present of the right arm and leg, which he moved with difficulty. Complete aphasia present. He could neither articulate nor did he appear to understand what was said to him. Temperature, normal ; pulse, 96. ℞. Ice to the head ; iodide potash, gr. x.; fluid ext. ergot, mm. xx., every four hours.

On the following day, November 14th, he appeared somewhat better, could raise his arm and leg at will, although with difficulty, and seemed to understand questions and to make an attempt to answer, but he was unable to utter a sound. He was restless and sleepless ; pulse, 120. After consultation with Dr. Crego, the neurologist to this hospital, who concurred in the diagnosis of a clot on the brain, either extra- or intradural, over the center of speech, extending upwards and involving the motor area on the left side, operation was decided upon and performed on the following day, November 16th, after the usual antiseptic precautions had been taken. A curved incision, convex downwards, was made from the external lower border of the frontal bone downwards and then upwards, terminating just above the ear. The large wings of the sphenoid and the temporal bone were exposed, and by aid of a large trephine—one and one-quarter inches in diameter—a button of bone removed over the base of the third frontal convolution, involving the center of speech and face. No extradural bleeding was found. The dura was bluish in color, and bulged forwards, showing great pressure behind. No pulsation was seen. A small puncture was made through the dura, through which a fine stream of old blackish blood spurted several feet in the air. The dura was, therefore, freely opened, and a large amount of similar blood, estimated at three ounces, evacuated. A large bloodclot now appeared, covering an area of about three square inches and being about one-quarter inch thick. It extended upward and backward, was firmly adherent, and was removed with difficulty, partly by aid of forceps, partly by sharp spoon. Pulsation of the brain was thereafter plainly seen. The brain seemed excavated and pressed inward where the clot had been removed. The wound was thereafter thoroughly irrigated with warm sterilized water, the dura sutured with fine catgut, the button replaced, the wound closed without drainage, and antiseptic dressings applied.

The further course was very favorable. The wound healed by first intention. Two days afterward, he could say yes and no and count to four; two days later the aphasia had disappeared and the patient was able to talk freely, although pronouncing some words with hesitation and difficulty. The paresis of the arm and leg disappeared completely in a week, and the patient got up. On his discharge, four weeks later, it was noted that vocal fremitus over the left side of the head was normal, the pupils normal in size and reaction. Paralysis of the face had entirely disappeared, and the condition of the facial nerve was normal. He was able to draw the face to either side and raise the nostril. The tongue could be protruded without deviation to either side. His right hand was somewhat weaker, registering 70 to left 90. Knee reflexes normal; muscular power and sensibility perfect. Aphasia had completely disappeared; memory was perfect, and he left the hospital mentally and physically completely restored.

His assailant was discharged from jail a wiser, and, I hope, a more peaceable man, a week after the operation.

This case is of interest on account of the correct diagnosis verified by prompt operation, the replacement and successful implantation of the large button, by which adhesions of dura to the skin and possible future troubles were avoided, the rapid disappearance of all the serious symptoms, and the complete restoration of the patient to health, and of his assailant to liberty. I wish here to call attention to the dangers of using strong antiseptics in operations on the brain. Physiological investigations have shown, as published in *Deutsche Medicinische Wochenschrift*, that the brain is extremely sensitive to chemical irritants, and it was found that carbolic acid, in strength above 1 to 200, speedily produced death, and that corrosive sublimate, even as weak as 1 to 10,000, inflicted severe injury upon the brain tissue. I believe that nothing but sterilized water ought to be used in brain injuries or operations.

### TREPHINING FOR ABSCESS OF BRAIN—RECOVERY.

Case XIII.—G. B., 4 years of age, entered the Sisters' hospital on November 22, 1893. Eight weeks previously, he had been injured on the top of his head by a "rasp," which some other boys were throwing up in the air. The injury consisted of a punctured wound of scalp and skull. No physician was called, the wound was bandaged at home, and the little boy continued playing. The next day the head was swollen and the boy remained in bed all day, but thereafter he apparently got well, and continued healthy for six weeks. Two weeks ago he began to get easily fatigued, looked pale, had slight fever at night, attacks of vomiting, and increasing drowsiness and stupor. One week ago, the family physician was called, found the boy with a fever of 103, rapid pulse, mental dullness, and stupor. He advised his removal to the hospital, but the parents first consented one week later, as he was rapidly growing worse and the stupor was increasing.

On examination, he looked pale, with heavily-coated tongue, slight facial paralysis on left side, otherwise no symptoms of paresis. He was in a state of stupor, fretful and peevish, and could not, or would not, answer any questions. A small scar was seen on the top of his head, to the right of the median line and just behind the coronal suture. Temperature 103; pulse 130. The symptoms indicated a brain abscess, and, after consultation with Dr. Crego, exploratory trephining was advised and performed immediately.

A downward convex incision was made, circumscribing the scar. After the periosteum had been removed, a small opening, filled with granulations, was seen in the skull. A half-inch button was removed with the trephine, outside the hole, and a rim of bone removed around the hole with cutting forceps (Keen's). The dura mater thereafter bulged into the opening, was not pulsating, and a granulating mass found, corresponding to the hole in the skull. The dura was opened, and just beneath the granulations a stream of pus gushed out from an abscess cavity beneath the cortical substance. The opening was enlarged with dressing-forceps and about two ounces of pus evacuated. The cavity seemed, by exploration with the finger, to be about the size of a large egg. It was irrigated with sterilized water, loosely packed with iodoform-gauze, let out through an opening in the flap corresponding to the deeper opening. The wound was thereafter sutured and antiseptic dressing applied.

November 23d. He rested quietly during the night and seems much brighter, says he feels "first rate," and wishes to get up. Pulse, 120; temperature, 101.

November 24th. Wound dressed, irrigated and iodoform again introduced.

November 26th. Temperature, 99. The wound was thereafter irrigated and dressed every other day, a small strip of iodoform gauze being introduced for drainage; the pus discharge diminished rapidly, and he left the hospital, convalescent, on December 10th.

From a letter from his physician, of January 16, 1894, I quote: "Mentally and physically the child seems to be perfectly well has gained in weight, and is as active as ever. The discharge of pus ceased shortly after his leaving the hospital, and the wound is nearly healed."

The interest in this case is the entire absence of paralysis, although the abscess, evidently, was near the motor center and must have exerted considerable pressure on this point.

The diagnosis was made on the symptoms of mental dullness and elevation of temperature.

For the history of the following case I am indebted to Dr. W. C. Krauss, of Buffalo:

### TREPHINING FOR EPILEPSY.—RECOVERY.

CASE XIV.—W. T., of Attica, N. Y.; age, 24; height, five feet eight inches; weight, 165 pounds; constitution strong and healthy. Nothing in his antecedents and early life is worthy of special consideration. When twelve years of age an accident befell him, on a Fourth of July, which proved to be the cause of his later misfortune. Some boys having procured the barrel of an old gun, were engaged in discharging it to help celebrate our national holiday. The gun flew into the air and, on descending, struck the patient on the left frontal region of the head. He was carried unconscious to a physician's office, who removed several small bone particles of the skull and sewed up the wound. Suppuration set in, which continued for a few weeks, and the boy seemed to have recovered fully from the effects of the injury. About six years ago, six years after the accident, he began to notice that he felt unusually drowsy, dull, and stupid on awakening in the morning. This condition lasted for some time, the cause undiscovered, until he was discovered in an epileptic attack by his room-mate.

He consulted several physicians without obtaining any relief. In the Fall of 1890, he consulted Dr. Krauss, who, without any success, prescribed bromides. He made a thorough examination and found a large deep depression over the left frontal bone, the posterior edge extending through the coronary suture. Up to this time he had been able to work hard on a farm, but would have the nocturnal attacks.

On May 2, 1891, Dr. Krauss was called to Attica to see him, and found him in one of the attacks. He was sitting in a chair, his eyes wide open, glassy, staring, his head drawn to the left, speechless, but not exhibiting any signs of convulsions.

These attacks were growing more frequent, had become diurnal as well nocturnal, and some radical procedure was demanded. Two days later, Dr. Krauss was again called to Attica, and I was requested to accompany him and operate. On our arrival, we found the patient in the status epilepticus, the attacks being no longer intermittent but continuous, without longer intervals than a couple of minutes. He had been in this condition for thirty hours, one attack of convulsions following the other without the patient regaining consciousness. The pulse was weak and rapid, face cyanotic. A large depression, covered with a tight, whitish, adherent scar, was seen, as described. A large curved incision, convex backwards, was made, the scalp loosened with difficulty from the bone, and an irregular defect of bone found under the scar. The surrounding edges of the bone were much hypertrophied, strongly adherent to the dura and pressing it inwards. The dura was loosened all around from the hypertrophied bone, which thereafter was removed all around the defect with cutting forceps.

During the following day he had six attacks, but thereafter he improved rapidly, and he soon returned to his work a well man. No attacks occurred for seventeen months, he got married and felt well, but continued the treatment with bromides. On October 7, 1892, he consulted Dr. Krauss again, and stated that the attacks were returning and were becoming daily more frequent. Dr.

Krauss advised him to enter the Sisters' hospital, in Buffalo, in order that he could be under observation and treatment. The attacks, similar to the ones he had at first, grew daily more frequent and intense, and after three days he was in the same condition of status epilepticus. An operation, similar to the first, but convex forwards, where the bone could not be reached during the first operation, was performed, hypertrophied bony edges, strongly adherent to dura mater, found and removed and the dura again widely loosened from the bone with Horsley's spatula. He recovered rapidly, having some attacks only during the following twenty-four hours, and was discharged from the hospital on November 6, 1892, apparently cured. He was again treated by Dr. Krauss with bromides, his food restricted, and all work prohibited for six months. Thereafter he resumed his former work. He has since had no more attacks or peculiar feelings, his physical and mental condition, Dr. Krauss states, are excellent, and we hope for no further trouble. The depression is covered by his hair and gives him no concern whatever.

The interest in this case is the late appearance after the injury, the apparent recovery after the first operation, relapse, and a still further remission and a possible cure after a second operation. It is the belief of Dr. Krauss and Dr. Crego, who saw him the second time, that death promptly would have occurred if he had not been operated on.

